# Reevaluation of Lung Injury in TNF-Induced Shock: The Role of the Acid Sphingomyelinase

**DOI:** 10.1155/2020/3650508

**Published:** 2020-05-01

**Authors:** Lucy K. Reiss, Ute Raffetseder, Lydia Gibbert, Hannah K. Drescher, Konrad L. Streetz, Agatha Schwarz, Christian Martin, Stefan Uhlig, Dieter Adam

**Affiliations:** ^1^Institute of Pharmacology and Toxicology, Medical Faculty of RWTH Aachen University, Wendlingweg, 52074 Aachen, Germany; ^2^Division of Nephrology and Clinical Immunology, Medical Faculty of RWTH Aachen University, Pauwelsstr. 30, 52074 Aachen, Germany; ^3^Gastrointestinal Unit and Liver Center, Massachusetts General Hospital, Harvard Medical School, Boston, MA 02114, USA; ^4^Department of Medicine III, Medical Faculty of RWTH Aachen University, Pauwelsstr. 30, 52074 Aachen, Germany; ^5^Department of Dermatology, Christian-Albrechts University, Arnold-Heller-Str. 3, 24105 Kiel, Germany; ^6^Institute of Immunology, Christian-Albrechts University, Arnold-Heller-Str. 3, 24105 Kiel, Germany

## Abstract

Tumor necrosis factor (TNF) is a well-known mediator of sepsis. In many cases, sepsis results in multiple organ injury including the lung with acute respiratory distress syndrome (ARDS). More than 20-year-old studies have suggested that TNF may be directly responsible for organ injury during sepsis. However, these old studies are inconclusive, because they relied on human rather than conspecific TNF, which was contaminated with endotoxin in most studies. In this study, we characterized the direct effects of intravenous murine endotoxin-free TNF on cardiovascular functions and organ injury in mice with a particular focus on the lungs. Because of the relevance of the acid sphingomyelinase in sepsis, ARDS, and caspase-independent cell death, we also included acid sphingomyelinase-deficient (ASM^−/−^) mice. ASM^−/−^ and wild-type (WT) mice received 50 *μ*g endotoxin-free murine TNF intravenously alone or in combination with the pan-caspase inhibitor carbobenzoxy-valyl-alanyl-aspartyl-[O-methyl]-fluoromethylketone (zVAD) and were ventilated at low tidal volume while lung mechanics were followed. Blood pressure was stabilized by intra-arterial fluid support, and body temperature was kept at 37°C to delay lethal shock and to allow investigation of blood gases, lung histopathology, proinflammatory mediators, and microvascular permeability 6 hours after TNF application. Besides the lungs, also the kidneys and liver were examined. TNF elicited the release of inflammatory mediators and a high mortality rate, but failed to injure the lungs, kidneys, or liver of healthy mice significantly within 6 hours. Mortality in WT mice was most likely due to sepsis-like shock, as indicated by metabolic acidosis, high procalcitonin levels, and cardiovascular failure. ASM^−/−^ mice were protected from TNF-induced hypotension and reflex tachycardia and also from mortality. In WT mice, intravenous exogenous TNF does not cause organ injury but induces a systemic inflammatory response with cardiovascular failure, in which the ASM plays a role.

## 1. Introduction

Despite a multitude of experimental and clinical studies, the exact inflammatory mechanisms of the acute respiratory distress syndrome (ARDS), a life-threatening respiratory failure, and the frequently resulting multiple organ failure (MOF) remain unknown. The cytokine tumor necrosis factor (TNF) is generally thought to play a pivotal role in the pathogenesis of sepsis and ARDS [1, 2], which is frequently caused by sepsis [3, 4]. This view is based on (i) early experimental and clinical studies, which have shown that infusion of human TNF into experimental animals induced symptoms of septic shock, including fever, cardiovascular impairment, and organ failure involving the lungs, kidneys, and liver [5–8]; (ii) elevated TNF levels in the bronchopulmonary secretions of patients with ARDS [9], which have been correlated with a poor outcome [10]; and (iii) improved survival in patients subjected to anti-TNF treatment in clinical sepsis trials [11]. Nonetheless, in spite of the great attention that TNF has received, surprisingly, little is known about the immediate effects of circulating TNF on the lung and other organs. In more than 20-year-old studies, which examined the pulmonary effects of human TNF *in vivo*, increased microvascular permeability with subsequent pulmonary edema and acute lung injury were reported [7, 12–14]. The fact that these early findings have not been confirmed with conspecific TNF as well as the more recent availability of murine endotoxin-free TNF led us to reevaluate the effects of intravenously (i.v.) injected TNF in mice.

The enzyme acid sphingomyelinase (ASM) is another important mediator in the pathogenesis of sepsis and ARDS [15]. Sepsis patients exhibited increased levels of secretory ASM activity, and the ASM serum activity was shown to be a useful predictor for mortality in sepsis [16, 17]. In endotoxemia and in models of intrapulmonary ARDS, pharmacological blocking of ASM and ASM deficiency protected against vascular barrier disruption [18–20] and also reduced mortality due to septic shock [16, 18]. Several mechanisms were proposed by which the promotion of pulmonary edema is linked to the ASM [21], particularly when TNF is present [22]. It is well established that TNF activates the ASM [18, 23, 24], which was the rationale for including ASM-deficient (ASM^−/−^) mice in this study, which we expected to be protected from TNF-induced injury.

In addition to its critical role in immune reactions, TNF can induce caspase-dependent and caspase-independent cell death. Interestingly, in mice, the toxicity of TNF was shown to be aggravated by pan-caspase inhibition with carbobenzoxy-valyl-alanyl-aspartyl-[O-methyl]-fluoromethylketone (zVAD), resulting in hyperacute shock with increased mortality and renal failure [25, 26]. In the current study, we aimed to further investigate the caspase-independent effects of TNF with the use of zVAD. We presumed that this would aggravate TNF-toxicity and result in organ failure.

The objective of the current study was to use a high dose of highly purified conspecific TNF to investigate the direct effects of TNF on organ injury and cardiovascular functions with a particular emphasis on the lung. In order to delay lethal shock and to obtain clinically relevant results, mice were ventilated in a “mouse intensive care unit” (MICU), which facilitates monitoring and stabilization of body temperature and blood pressure for several hours [27, 28]. The delay of hypovolemic shock by fluid support was intended to gain time for the development of organ failure.

In line with our hypothesis, TNF caused a systemic inflammatory response including lethal cardiovascular failure, which was absent in the ASM^−/−^ mice. Surprisingly, however, TNF—despite being highly effective in producing shock—failed to cause significant lung, kidney, or liver injury in WT mice.

## 2. Methods

### 2.1. Mice

Experiments were performed with female and male C57BL/6x129/SvEv wild-type (WT) and ASM-deficient (ASM^−/−^) mice aged 8 to 10 weeks, weighing 25 to 30 grams. The experimental protocols were in accordance with the German animal protection law and approved by regional governmental authorities (Landesamt für Natur, Umwelt und Verbraucherschutz NRW, permission number AZ 8.87-50.10.37.09.287).

### 2.2. Surgical Procedures and Mechanical Ventilation

Mice were anesthetized intraperitoneally with pentobarbital sodium (75 mg/kg) and fentanyl (40 *μ*g/kg) and ventilated with low tidal volume (*V*_T_) as described [27]. Lung functions were followed by the low-frequency forced oscillation technique (flexiVent, SCIREQ, Montreal, Canada). After 30 min of ventilation, baseline values were reached and 100 *μ*L NaCl was injected i.v. to stabilize cardiovascular functions. Another 15 min later, mice received 50 *μ*g of endotoxin-free (<0.1 ng/*μ*g, <1 EU/*μ*g) murine TNF (PeproTech, Rocky Hill, USA) in 100 *μ*L of NaCl followed by 6 h of ventilation. In addition, two groups were treated with the general caspase inhibitor zVAD (Bachem AG, Bubendorf, Switzerland), which was suspended in DMSO at 125 mg/mL and further diluted with 0.9% NaCl to 2.5 mg/mL. Based on the study by Cauwels et al. [25], zVAD was injected i.v. twice: 250 *μ*g zVAD after 30 min of ventilation in 100 *μ*L of 0.9% NaCl and 100 *μ*g zVAD together with 1 mg bovine serum albumin (BSA) after 60 min of ventilation in 140 *μ*L total volume (for experimental design, see Supplemental Fig. 1). The following groups were investigated: TNF alone (ASM^−/−^+TNF (*n* = 5) and WT+TNF (*n* = 5)) and TNF and zVAD (ASM^−/−^+zVAD/TNF (*n* = 5) and WT+zVAD/TNF (*n* = 5)); control groups were ventilated for 405 min and received saline or saline+DMSO instead of TNF or zVAD, respectively (ASM^−/−^ sham (*n* = 6) and WT sham (*n* = 5)). A catheter for measurement of the mean arterial pressure (MAP) and fluid support was inserted into the carotid artery. In order to stabilize blood pressure, 0.9% NaCl (200 *μ*L/h) was infused intra-arterially (i.a.) and body temperature was stabilized at 37°C (homeothermic blanket, Harvard Apparatus Holliston, MA, USA). The heart frequency (HF) was calculated from the electrocardiogram (PowerLab, ADInstruments, Spenbach, Germany). Mice were sacrificed by exsanguination via the arterial line. Blood samples were analyzed by blood gas analysis (ABL700, Radiometer, Copenhagen, Denmark). Lungs were dissected, lavaged, and processed as described [27].

### 2.3. Enzyme-Linked Immunosorbent Assays

Proinflammatory mediators, BSA, and procalcitonin (PCT) were quantified in plasma and BAL fluid with enzyme-linked immunosorbent assays (ELISA) (mediators: R&D Systems, Abingdon, UK; BSA: Cygnus Technologies, Southport, USA; and PCT: Uscn, Wuhan, China). The ratio of BSA in plasma and BAL fluid was calculated to determine microvascular permeability. Cell death was assessed by quantifying mono- and oligonucleosomes by ELISA (Roche Applied Science, Mannheim, Germany). The assay was performed with 30 mg of homogenized frozen lung tissue according to the manufacturer's instructions. The lysis buffer was measured as blank, which was subtracted from the results. The cell death ratio was calculated as % per mg lung tissue relative to a 100% positive control provided with the assay.

### 2.4. Histopathological Analyses

The left lung was filled with 4% buffered formaldehyde. The lung, kidneys, and part of the median lobe of the liver were fixed in 4% buffered formaldehyde and embedded in paraffin. The lungs and livers were cut in sections of 3 *μ*m followed by hematoxylin and eosin (HE) staining. For the lung, a scoring system for experimental lung injury was used [29]. 10 fields per lung were evaluated according to the following criteria: A: neutrophils in the alveolar space, B: neutrophils in the interstitial space, C: hyaline membranes, D: proteinaceous debris in the airspaces, and E: alveolar septal thickening. The score was calculated as follows: Score = [(20 × A) + (14 × B) + (7 × C) + (7 × D) + (2 × E)]/(number of fields × 100). The kidneys were sectioned at 1 *μ*m, stained with periodic acid-Schiff (PAS), and counterstained with hematoxylin. The tubular injury was scored on a scale of 0–4: 0 = normal histology, 1 = mild dilatation, 2 = flattened epithelial cells and loss of brush border, 3 = mild vacuolization of tubular cytoplasm (<5 vacuoles/tubule) and tubular cell necrosis/apoptosis, and 4 = strong vacuolization of tubular cytoplasm (>5 vacuoles/tubule) and tubular cell necrosis/apoptosis. The total score is the calculated average of all tubular scores. A total of 100 tubuli per section were evaluated. Histopathology was evaluated in a blinded manner.

### 2.5. TUNEL Staining

TUNEL staining (Roche) was performed according to the manufacturer's instructions with 1 *μ*m paraffin kidney sections. Quantification of TUNEL staining was performed by counting the positively stained nuclei in 20 randomly selected visual fields at original magnification ×100 in the renal cortex.

### 2.6. Western Blot Analysis

Denatured urine samples were separated by electrophoresis in 10% SDS-PA gels, transferred to nitrocellulose membranes, blocked with 2% BSA in Tween Tris-buffered saline (TTBS) buffer, washed with TTBS, and incubated with a rabbit polyclonal anti-kidney injury molecule- (KIM-) 1 antibody (Acris, San Diego, USA) diluted to 1 : 1000 in TTBS overnight at 4°C. The KIM-1 antibody was detected using a HRP-conjugated goat anti-rabbit IgG (Dako, Hamburg, Germany) antibody and was visualized by enhanced chemiluminescence.

### 2.7. Nitric Oxide Quantification

A nitric oxide (NO) quantification assay (R&D Systems, Abingdon, UK) was used to measure total NO in blood plasma samples. For this purpose, endogenous nitrate was converted to nitrite by nitrate reductase. The total nitrite was quantified as a chromophoric azo-derivative of the Griess reagent at 550 nm.

### 2.8. Statistical Analyses

Time courses were analyzed by the mixed model procedure. The Bartlett test was used to check for equal variances. Box-Cox transformation was performed to achieve homoscedasticity, when suitable. Normal distribution of the residuals was validated with the Shapiro-Wilk test. Univariate data were analyzed with one-way analysis of variance, followed by the *t*-test and FDR correction or the Tukey posttest. Percentage data were transformed using the arcsine transformation prior to analysis. Nonparametric data, except for survival curves, were analyzed with the Kruskal-Wallis test followed by Dunn's posttest. Survival curves were compared with the Kaplan-Meier estimator. For this analysis, the groups TNF and zVAD/TNF, which showed no difference in survival, were pooled for each genotype, resulting in *n* = 10. Correlation was evaluated with Pearson's correlation coefficient. Statistical analyses were carried out with GraphPad Prism 5.0 (GraphPad Software, La Jolla, USA) or SAS 9.4 software (SAS Institute, Cary, USA). A *p* value < 0.05 was considered significant. Data are shown as mean + SEM with *n* = 6 in the group ASM^−/−^ sham and *n* = 5 in all other groups. In all experiments, posttests were performed by comparing equally treated groups of different genotypes as well as all groups within the same genotype with each other.

## 3. Results

### 3.1. Survival

**Figure 1:**
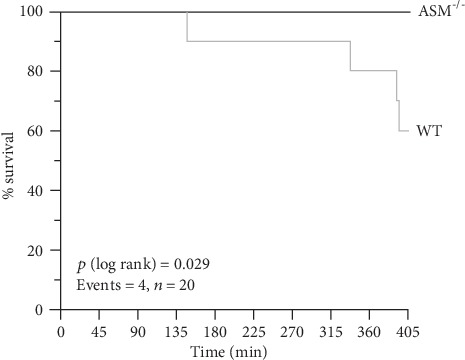
Survival. Survival was compared by univariate Kaplan-Meier analysis in TNF-treated WT and ASM^−/−^ mice. Four WT mice died during the experiment, whereas all TNF-treated ASM^−/−^ mice survived (*p* < 0.05, *n* = 10 per group).

Despite of the mechanical ventilation, the stabilization of body temperature at 37°C, and the intravascular volume support of around 1500 *μ*L in total over 405 min (see Supplemental Fig. 1 for experimental design), four WT mice died in response to TNF, whereas all the ASM^−/−^ mice survived (Figure 1). Thus, genetic depletion of ASM confers significant protection from TNF-induced mortality. Next, we analyzed whether the TNF-induced mortality in WT mice can be explained by organ failure.

### 3.2. Effects of TNF on the Lung

**Figure 2:**
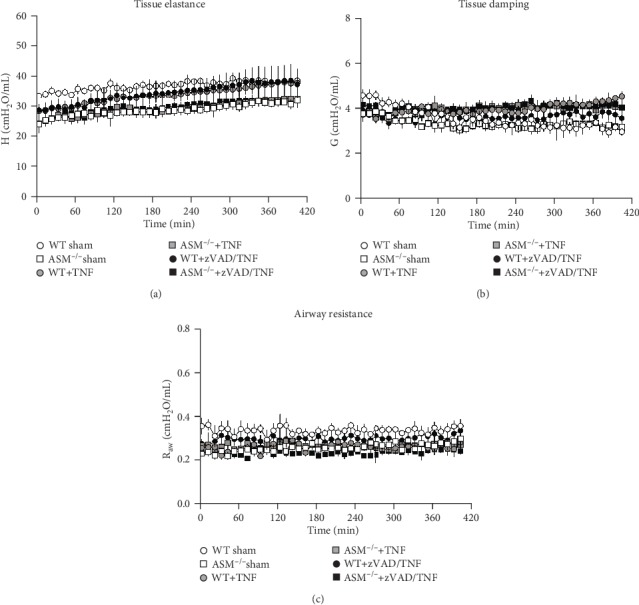
Lung mechanics. (a) Tissue elastance (H), (b) tissue damping (G), and (c) airway resistance (R_aw_) were measured by the forced oscillation technique every ten minutes. Data are shown as mean ± SEM with ASM^−/−^ sham *n* = 6 and *n* = 5 in all other groups.

Measurement of the pulmonary input impedance revealed that TNF did not alter lung mechanics. Lung tissue elastance (H) was in a physiologically normal range in all groups (Figure 2(a)). For unknown reasons, baseline values of H were slightly higher in WT sham than in ASM^−/−^ sham mice (*p* < 0.001), but there was no significant increase in elastance due to TNF or TNF+zVAD. Tissue damping (G) (Figure 2(b)) and airway resistance (R_aw_) (Figure 2(c)) remained unchanged and also in a normal range in all groups, further indicating that high systemic TNF levels had no injurious effects on the lung. Biochemical assessment of ASM activity in the lung tissue confirmed that ASM^−/−^ mice had no functional ASM (Supplemental Fig. 2).

The pO_2_/FiO_2_ ratio of around 500 mmHg (66.7 kPa) and the mean pCO_2_ of around 38 mmHg (5.1 kPa) indicated unimpaired gas exchange in all experimental groups and provided further evidence that TNF did not harm the lung (Table 1).

**Figure 3:**
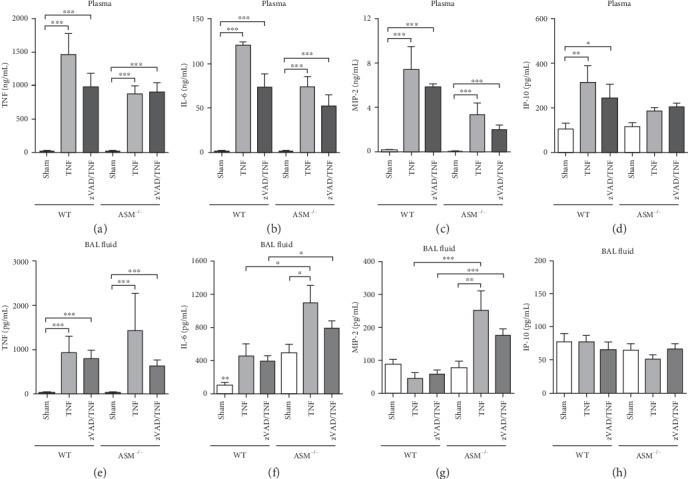
Proinflammatory mediators. Levels of TNF, IL-6, MIP-2, and IP-10 quantified by ELISA (a–d) in the blood plasma and (e-h) in bronchoalveolar (BAL) fluid, which was taken from the right lung after the ventilation experiment. Data are shown as mean + SEM with *n* = 6 in the group ASM^−/−^ sham and *n* = 5 in all other groups. ^∗^*p* < 0.05, ^∗∗^*p* < 0.01, and ^∗∗∗^*p* < 0.001.

TNF was quantified in the blood plasma to examine the distribution of the i.v. injected TNF in the circulation. High TNF concentrations of around 1000 ng/mL plasma were detected in all TNF-treated mice (Figure 3(a)). Notably, also the BAL fluid contained high TNF levels (Figure 3(e)), showing that the intravenously injected TNF had entered the lungs. In the control groups ASM^−/−^ and WT sham, plasma and BAL TNF levels were near the detection limit.

IL-6 and MIP-2 plasma concentrations were strongly elevated by TNF in all groups compared to the control groups (Figures 3(b) and 3(c)), whereas IP-10 (CXCL10) liberation was only clearly increased in the blood of WT mice (Figure 3(d)). In the BAL, IL-6 levels (Figure 3(f)) were only moderately increased by TNF and in contrast to the plasma values, with higher concentrations in ASM^−/−^ than in WT mice. TNF increased MIP-2 (CXCL2) BAL levels only in ASM^−/−^ mice (Figure 3(g)). IP-10 BAL concentrations were equally low in all groups (Figure 3(h)). zVAD did not affect the liberation of the analyzed mediators. Interestingly, MIP-2 and IP-10 concentrations were higher in the plasma than in the BAL in all TNF-treated groups, indicating that the chemotactic gradients induced by TNF were directed towards the vascular side.

**Figure 4:**
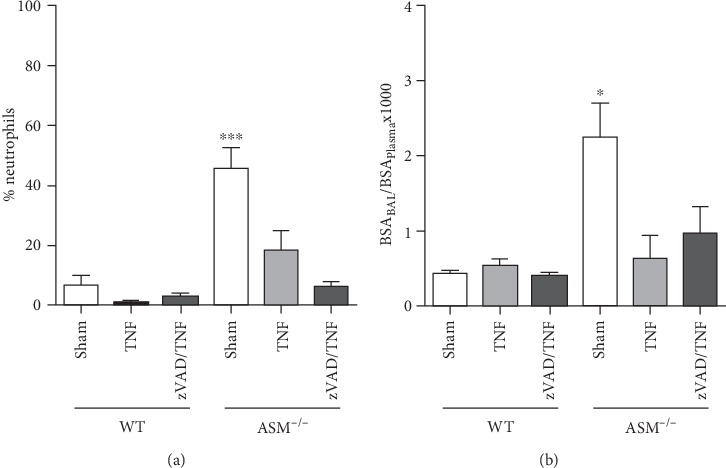
Microvascular permeability. (a) Intra-alveolar neutrophils were assessed by cytospin preparation of the bronchoalveolar lavage (BAL) fluid. (b) Bovine serum albumin (BSA) was injected into the tail vein after 105 min of ventilation. The ratio between BAL and plasma BSA levels is shown as an indicator for microvascular permeability. Data are shown as mean + SEM with *n* = 4 in the group WT sham, *n* = 6 in ASM^−/−^ sham, and *n* = 5 in the other groups. ^∗^*p* < 0.05, ^∗∗∗^*p* < 0.001.

Although neutrophil chemotactic factor MIP-2 was strongly increased in BAL of ASM^−/−^ compared to WT mice, no extravascular migration was observed following TNF application. Thus, quantification of intra-alveolar neutrophils revealed that these were not recruited in response to TNF or TNF+zVAD (Figure 4(a)). In contrast, the relatively high numbers of neutrophils in the lungs of ASM^−/−^ mice were strongly reduced following TNF injection. In line with this, microvascular permeability, which was measured as albumin influx into the alveoli, was higher in ASM sham than in WT mice and was reduced in TNF-treated ASM^−/−^ mice (Figure 4(b)).

**Figure 5:**
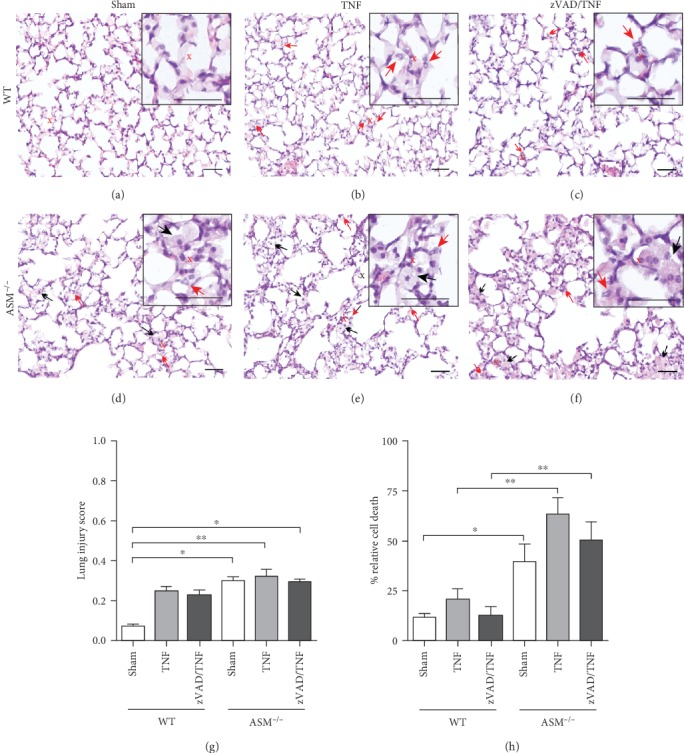
Lung histopathology and cell death. Representative HE-stained sections from (a–c) WT mice and (d–f) ASM^−/−^ mice, treated as indicated above the image. Scale bars 50 *μ*m, magnification 200x. Enlarged sections are shown in the upper right corners (scale bars 50 *μ*m). The center is indicated by a red cross in the original image. Red arrows indicate interstitial neutrophils; black arrows indicate foam cells. (g) The lung injury score was assessed according to [29] by evaluating 10 fields per lung regarding the following criteria: neutrophils in the alveolar space, neutrophils in the interstitial space, hyaline membranes, proteinaceous debris in the airspaces, and alveolar septal thickening. Data are shown as mean + SEM with *n* = 6 in the group ASM^−/−^ sham and *n* = 5 in all other groups. ^∗^*p* < 0.05, ^∗∗^*p* < 0.01. (h) Cell death was quantified in homogenized lung tissue using an ELISA against mono- and oligonucleosomes and is shown as % per mg lung tissue relative to a 100% control provided with the assay. Data are shown as mean + SEM with WT sham (*n* = 5), WT+TNF (*n* = 5), WT+zVAD/TNF (*n* = 4), ASM^−/−^+TNF (*n* = 5), ASM^−/−^ sham (*n* = 6), and ASM^−/−^+zVAD/TNF (*n* = 5). ^∗^*p* < 0.05, ^∗∗^*p* < 0.01.

Histopathological examination further demonstrated that TNF had no significant effect on the lungs of WT (Figures 5(b) and 5(c)) and ASM^−/−^ mice (Figures 5(e) and 5(f)), albeit the injury score was increased by around 10% in all TNF-treated groups. This increase is due to a slight increase in the number of interstitial neutrophils (indicated by red arrows) by TNF, but apart from this, no major alterations were observed. In ASM^−/−^ mice, alveolar obstructions with foamy enlarged macrophages filled with cytoplasmic lipid inclusions were present (indicated by black arrows), which increased the injury score in ASM^−/−^ sham compared to WT sham mice insignificantly (Figure 5(g)) and indicated the onset of sphingolipidosis.

In order to assess whether TNF had an impact on cell death in the lung, mono- and oligonucleosomes were quantified in lung tissue. Interestingly, in WT mice, the high pulmonary TNF levels had no significant influence on cell death in the lung (Figure 5(h)). Consequently, mice that had received TNF in combination with the pan-caspase inhibitor zVAD showed no injurious effects on the lung either. Furthermore, in ASM^−/−^ mice, relative cell death was only slightly increased by TNF, whereas it was clearly higher in all ASM^−/−^ groups compared to the corresponding WT mice. In addition, we performed an anti-cleaved caspase-3 immunostaining, which confirmed ongoing cell death in the lungs from ASM^−/−^ mice, even without TNF treatment, whereas the WT sham group was completely negative (Supplemental Fig. 3).

### 3.3. Effects of TNF on the Kidney and Liver

**Figure 6:**
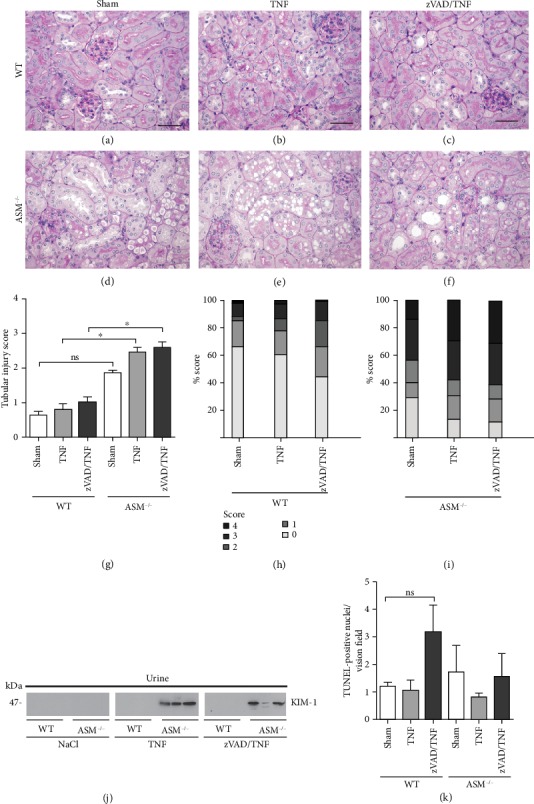
Kidney injury. Representative PAS-stained kidney sections from (a–c) WT mice and (d–f) ASM^−/−^ mice treated as indicated above; scale bars 50 *μ*m, magnification 400x. (g) The total tubular injury score. The tubular injury was scored in (h) WT and (i) ASM^−/−^ mice on a scale of 0–4: 0 = normal histology, 1 = mild dilatation, 2 = flattened epithelial cells and loss of brush border, 3 = mild vacuolization of tubular cytoplasm (<5 vacuoles/tubule) and tubular cell necrosis/apoptosis; 4 = strong vacuolization of tubular cytoplasm (>5 vacuoles/tubule) and tubular cell necrosis/apoptosis. (j) The tubular injury marker KIM-1 was analyzed in urine samples by western blot. Data are shown as mean + SEM with *n* = 5 in all groups except for ASM^−/−^ sham with *n* = 6. (k) TUNEL staining of representative kidney sections. ns: not significant, ^∗^*p* < 0.05.

Histopathology of PAS-stained kidneys displayed no major pathological alterations in WT mice, even after TNF treatment (Figures 6(a)–6(c)). In contrast, ASM^−/−^ sham mice showed distinct tubular vacuolization and sclerotic glomeruli (Figure 6(d)). Scoring of tubular injury confirmed that not only did ASM^−/−^ mice have a higher level of tissue damage, compared to WT mice, but also they were susceptible to injury by TNF (Figures 6(g)–6(i)).The protein kidney injury molecule- (KIM-) 1, which is an indicator for tubular injury, was detected in the urine from mice in the group ASM^−/−^+TNF and was not found in WT mice. Figure 6(j) shows an exemplary western blot with urine from three mice per group. TUNEL staining of kidney sections did not indicate cell death (Figure 6(k)). zVAD had no effect on the kidneys.

**Figure 7:**
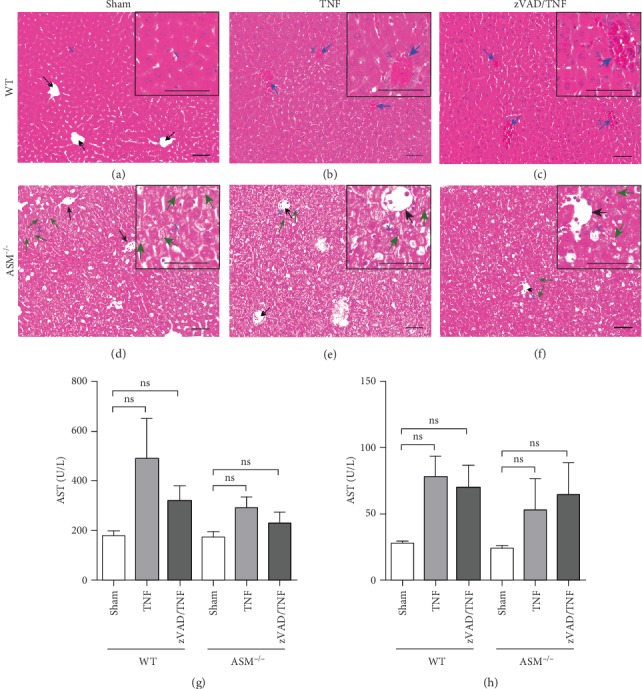
Liver injury. Exemplary HE-stained liver sections from (a–c) WT mice and (d–f) ASM^−/−^ mice treated as indicated above the image (scale bars 50 *μ*m, magnification 200x). Blue arrows indicate vascular congestion in WT; empty sinusoids are indicated by black arrows. Exemplary hepatocytes with granular cytoplasm and lipid inclusions are indicated by green arrows. The liver injury markers (g) aspartate aminotransferase (AST) and (h) alanine aminotransferase (ALT) were measured in the blood plasma. Data are shown as mean + SEM with *n* = 5 in all groups except for ASM^−/−^ sham with *n* = 6. ns: not significant.

Histological evaluation of the liver tissue revealed a normal morphology in WT sham mice (Figure 7(a)), whereas hemorrhage was evident in TNF-treated WT mice and sinusoids were clogged with red and white blood cells (indicated by blue arrows in Figures 7(b) and 7(c)), indicating blood stasis. Hemorrhage as well as blood-clogged sinusoids was absent in the livers of all ASM^−/−^ mice (open sinusoids indicated by black arrows) (Figures 7(d)–7(f)). The hepatocytes of ASM^−/−^ mice generally showed an altered morphology with granular cytoplasm, accentuated cell membranes, and intracellular lipid inclusions characteristic of Niemann-Pick disease. Exemplary cells are indicated by green arrows in Figure 7(d), whereby these alterations were apparent in the majority of hepatocytes. The blood plasma was analyzed for the liver injury markers aspartate aminotransferase (AST) and alanine aminotransferase (ALT), in which TNF increased to a minor and physiologically irrelevant extent (Figures 7(g) and 7(h)). Bilirubin levels were around 0.5 mg/dL in all mice and thus in the range of healthy animals (data not shown). Again, zVAD had no significant effect on organ injury.

### 3.4. Systemic Effects of TNF

**Figure 8:**
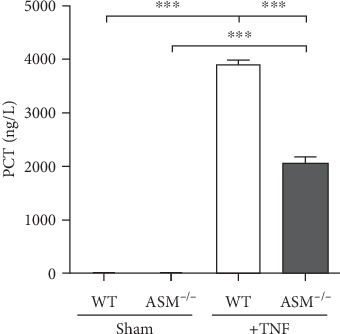
Procalcitonin in the blood plasma. Procalcitonin (PCT) was quantified by ELISA in blood plasma taken at the end of the experiment. PCT is an indicator for a sepsis-like condition. ^∗∗∗^*p* < 0.001.

Blood gas analysis showed that all TNF-treated mice suffered from severe metabolic acidosis with a pH < 7.2 (Table 1), indicating that TNF had caused sepsis-like symptoms. This was further supported by quantification of procalcitonin (PCT), a marker of sepsis and sterile inflammation, which revealed PCT levels above 2000 ng/L (Figure 8). Hemoglobin was increased in the group WT+TNF compared to the sham control and was higher in TNF-treated WT mice than in TNF-treated ASM mice (Table 1). zVAD had no systemic effect and is shown neither in this figure nor in the following figures.

**Figure 9:**
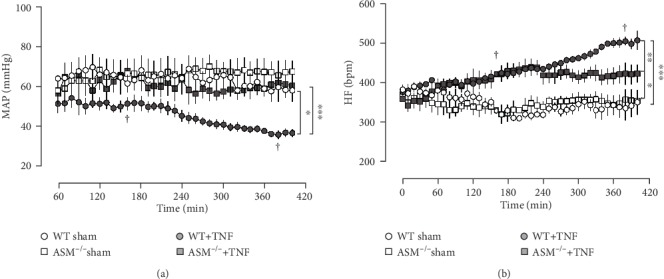
Cardiovascular parameters. (a) The mean arterial pressure (MAP) was measured in the carotid artery. (b) The heart frequency (HF) was calculated from the electrocardiogram. ^∗^*p* < 0.05, ^∗∗^*p* < 0.01, and ^∗∗∗^*p* < 0.001. Two mice died in the group WT+TNF, as indicated by †.

Mean arterial pressure (MAP) was generally low due to the anesthesia with pentobarbital but remained constant in controls (Figure 9(a)). In contrast, it descended over time in TNF-treated WT mice until it finally reached critically low values of <40 mmHg in survivors and <30 mmHg in nonsurvivors. The drop in blood pressure was accompanied by a continuous increase in heart frequency (HF) (Figure 9(b)). Interestingly, ASM^−/−^ mice appeared to be protected from the TNF-induced hypotension. The group ASM^−/−^+TNF showed no decline in MAP and only an initial moderate increase in HF.

**Figure 10:**
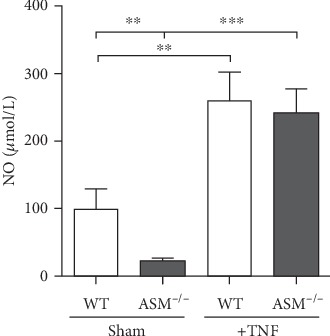
Total nitric oxide in the blood plasma. The stable nitric oxide (NO) metabolites nitrite and nitrate were quantified in the blood plasma, which was retrieved at the end of the experiment. ^∗∗^*p* < 0.01, ^∗∗∗^*p* < 0.001.

In this study, NO was quantified in the plasma to examine whether the refractory hypotension and impaired survival in WT mice could be related to vasodilatation due to an activation of the eNOS via the ASM. WT sham mice revealed significantly higher NO levels than ASM^−/−^ sham mice, and NO levels were significantly increased by TNF treatment, but there was no difference between TNF-treated ASM^−/−^ and WT mice (Figure 10).

Taken together, TNF caused a systemic inflammatory response including cardiovascular failure with lethal outcome exclusively in 40% of the WT mice, whereas ASM^−/−^ mice were protected against TNF-induced hypotension.

## 4. Discussion

This study was designed to reevaluate the effects of a high systemic dose of conspecific endotoxin-free TNF on pulmonary and cardiovascular functions and to investigate the caspase-dependent and caspase-independent contribution of the ASM therein. This study has two major findings. First, TNF alone is insufficient to cause pulmonary, renal, or hepatic injury. Second, we describe an important and previously unrecognized role for the ASM in hypotension resulting from a systemic inflammatory response.

### 4.1. The Effects of TNF on the Lung

The mortality in TNF-treated WT mice showed that the high dose of TNF, which was lethal in other studies before [18, 25, 30], had been effective (Figure 1). Despite this, lung injury did not occur (Figure 2 and Table 1). This surprising finding led to the question whether the injected TNF had actually entered the lung, which was proven by quantification of TNF in the BAL (Figure 3). The plasma levels of IL-6, MIP-2, and IP-10 mirrored the TNF distribution, which was distinctively higher in the plasma than in the BAL, indicating that the TNF injection had directed the chemotactic gradient towards the vascular side in both mouse strains. This hypothesis was supported by the observation that intra-alveolar neutrophil counts (Figure 4(a)) were in a range that can be regarded as normal for ventilated mice [27] and were not increased, but rather reduced by TNF, indicating that the neutrophils might have been held back in the circulation instead of sequestering in the lungs.

ASM^−/−^ mice had higher numbers of intra-alveolar neutrophils than equally treated WT mice, with the highest counts in the ASM^−/−^ sham group. Accordingly, this group showed signs of alveolar-capillary barrier disruption. Since neutrophil sequestration is not a common feature in ASM^−/−^ mice in general [31], the high numbers of neutrophils might have been induced by mechanical ventilation. Remarkably, TNF treatment did cause some protection in the lungs of ASM^−/−^ mice. In fact, microvascular permeability mirrored neutrophil sequestration showing that TNF did not cause neutrophil-independent edema (Figure 4(b)). This is consistent with the finding that TNF-induced edema was shown to be neutrophil-dependent in previous studies [32–34]. In line with this, TNF did not induce edema in isolated blood-free perfused rat or rabbit lungs [35] and intravenously applied TNF even protected from edema formation [36]. In contrast, in models where TNF was administered directly to the respiratory system, it triggered neutrophil recruitment and edema formation [37, 38]. Furthermore, in a small study with 17 patients, in which TNF was used for isolated limb perfusion, three patients developed respiratory failure secondary to lung edema [39], but it has to be considered that this trial was performed in cancer patients. The TNF-induced decrease in neutrophils and microvascular permeability in ASM^−/−^ mice indicates that these lungs were preinjured and further supports that TNF directed the chemotactic gradient towards the vascular side. It is noteworthy that TNF did not increase vascular permeability. Taken together, these findings strongly suggest that TNF had no direct effect on vascular permeability in the lungs.

In addition, the histopathological evaluation of the lungs (Figure 5) confirmed that the high concentration of circulating TNF did not generate pathological alterations, except for an increased number of interstitial neutrophils, which was also reported in an earlier study [40]. Histopathology further revealed that despite the young age of the mice (8-10 weeks), the lipid storage disease with formation of foam cells, typical of ASM deficiency [31], was already ongoing. This probably caused the increased BAL chemokine levels and most likely also explained the greater degree of cell death in ASM^−/−^ mice (Figure 5(h)). The presence of cleaved caspase-3-positive cells in the group ASM^−/−^+zVAD/TNF further supports that these cells were already apoptotic before the experiment (Supplemental Fig. 3).

One explanation for the lack of lung injury in this study might be the use of endotoxin-free TNF (<0.1 ng/*μ*g, <1 EU/*μ*g). In earlier experimental studies, in which TNF had an injurious effect, small amounts of endotoxin were present [7, 13, 14]. It is well known that TNF and endotoxin have a synergistic effect and that even small amounts of endotoxin increase the toxicity of TNF [41, 42]. Unfortunately, these studies had been performed without measurement of lung mechanics or oxygenation, so that it remains unanswered to which degree lung functions were actually impaired. Another difference of the current approach compared to the earlier ones is the use of murine TNF, which binds to both the murine TNF-R1 and TNF-R2 receptors and thus represents the physiologic ligand in the murine system, instead of the previously used human TNF, which binds selectively to the murine TNF-R1 [25]. The TNF-R1 promotes death signaling, pulmonary neutrophil sequestration, and edema formation, whereas the TNF-R2 was shown to protect against pulmonary edema [43] and to attenuate TNF-mediated inflammatory responses [44]. Therefore, TNF-R2 might have in part antagonized the inflammatory actions of the TNF-R1.

### 4.2. The Effects of TNF on the Kidney and Liver

Because TNF is known to play a role in renal and hepatic failure [5, 8, 45], kidneys (Figure 6) and livers (Figure 7) were also examined for organ injury. The lipid inclusions typical of ASM deficiency [31] found in the kidneys and the livers indicated that ASM deficiency itself had caused some organ damage, which is in line with our findings in the lung. Moreover, the kidneys of ASM^−/−^ mice were susceptible to injury by TNF, probably due to an increased liberation of proinflammatory mediators by migrated leukocytes, as shown in the lungs. Of note, TNF did not cause acute kidney injury (AKI) in WT mice (Figures 6(g)–6(j)), which is a frequent complication in sepsis [45]. In line with our findings, previous studies reported neither tubular or glomerular injury nor injury of other organs when TNF was used alone [25, 26]. This underlines the complexity of organ injury, which requires not one but a whole set of mediators as, e.g., released when lipopolysaccharide is used as stimulus [46, 47].

Finally, the blood-clogged sinusoids in the livers of WT mice indicated that these mice suffered from shock [48] (Figures 7(a)–7(c)). Interestingly, no pathological alterations of hepatic microcirculatory blood flow were evident in the livers of ASM^−/−^ mice, although these showed incipient manifestations of Niemann-Pick disease (Figures 7(d)–7(f)). The low TNF-induced increase of AST and ALT as well as unaltered low bilirubin values (data not shown) demonstrates that hepatic failure was not the cause of mortality in WT mice (Figures 7(g) and 7(h)). Previous studies with lower TNF doses infused over several days revealed an injurious potential of TNF [7, 49], and we cannot exclude that hepatocellular injury and failure might have developed after a longer period of time as consequence of the microvascular dysfunction [48]. In studies in which animals were not subjected to life support measures, high ALT levels were found already six hours after a lethal TNF challenge [40]. This illustrates the importance of a clinically relevant setting, which seems to critically affect the outcome in experimental sepsis models.

### 4.3. The Effects of zVAD

In the present study, the general caspase inhibitor zVAD had no ascertainable influence on the lungs, kidneys, or liver, which is most likely attributable to the circumstance that TNF did not disrupt the endothelium. As a result, zVAD stayed restricted to the vasculature. In addition, cell death played no relevant role here and therefore was not affected by zVAD. Thus, the use of zVAD did not allow a further characterization of the role of the ASM lung injury, despite the fact that our zVAD was active, as evidenced by a recent study in cancer cells in which we used the same lot of zVAD [50].

### 4.4. Cardiovascular Effects of TNF

Even though TNF failed to induce organ injury, it induced sepsis-like symptoms in both mouse strains. The severe metabolic acidosis in TNF-treated mice reflects an impaired microcirculation (Table 1). In addition, the high levels of PCT (Figure 8), a marker for sepsis in humans and mice [51], support that TNF induced a systemic inflammation and also show that the pathophysiological response to TNF is similar to that caused by infection. In both control groups, PCT levels lay below the detection limit, demonstrating that the increase in PCT was not caused by the operative interventions or the mechanical ventilation.

The clinical use of TNF as a drug in cancer therapy was found to be restricted by its toxicity, reflected in organ injury and hypotension [5, 8]. Refractory hypotension and reflex tachycardia, which can be regarded as manifestation of systemic inflammation [52], were observed also in the present study (Figure 9) and most likely caused death by hypoperfusion in WT mice (Figure 1). This is further supported by the observation that blood was centralized in the livers from WT mice, but not ASM^−/−^ mice as well as the higher hemoglobin levels in WT mice indicating plasma loss (Table 1). The fact that ASM^−/−^ mice were protected from hypotension and mortality, within the given time frame, indicates a novel role for the ASM in the pathogenesis of shock. This is in line with the finding that increased ASM levels correlated with the mortality of sepsis patients [16]. Interestingly, ASM^−/−^ mice were shown to be also resistant to endotoxin-induced mortality in an earlier approach, where the mechanisms of TNF- and endotoxin-induced shock were largely identical [18]. However, the cardiovascular effects in TNF-induced shock were not addressed in that context.

One mechanism by which the ASM could be involved in the development of shock in response to TNF is the activation of NO synthases by ceramide, resulting in vasodilatation and hypovolemic shock, as it was already shown for the neutral sphingomyelinase and eNOS [22, 53, 54]. However, the measurement of total plasma NO revealed no differences between TNF-treated WT and ASM^−/−^ mice, although NO was clearly increased by TNF and also higher in WT sham than in ASM sham mice (Figure 10). It has to be taken into account that NO quantification can be affected by hemolysis [55], which was present particularly in the plasma samples of TNF-treated WT mice. Therefore, a participation of the ASM in NO liberation cannot be excluded completely. The mechanisms by which the ASM mediates the TNF-induced hypotension as well as possible direct effects of TNF on the heart, such as myocarditis, which was shown to be lethal in TNF-overexpressing mice [56], need to be investigated in the future.

In the present study, not only the fluid support but particularly the stabilization of body temperature may have influenced the outcome, as it prevented hypothermia, which corresponds with fever in mice, and might have prevented a more rapid death of TNF-treated mice. Despite these measures, the very high TNF dose (50 *μ*g) induced death more rapidly in the present study compared to the previous two studies in which 15-25 *μ*g TNF was used [25, 26], wherefore the experimental time frame was limited and might have restricted the development of organ injury.

## 5. Conclusion

We conclude from our data that circulating TNF alone is not sufficient to increase vascular permeability in the lungs or to cause organ injury, which probably requires additional mediators. The present study suggests that the ASM plays an important role in the development of TNF-induced hypotension and thereby promotes mortality. Therefore, pharmacological inhibition of the ASM as approach to prevent lethal cardiovascular failure in septic shock should be further investigated.

## Figures and Tables

**Table 1:** Blood gas results.

	WT sham	WT+TNF	WT+zVAD/TNF	ASM^−/−^ sham	ASM^−/−^+TNF	ASM^−/−^+zVAD/TNF
pO_2_/FiO_2_ (cmH_2_O)	503 ± 25	533 ± 22	516 ± 42	499 ± 37	484 ± 37	498 ± 48
pCO_2_ (cmH_2_O)	38 ± 2	38 ± 2	37 ± 8	36 ± 2	38 ± 7	41 ± 5
pH	7.35 ± 0.03^#^	7.07 ± 0.04	7.15 ± 0.05	7.38 ± 0.02^#^	7.16 ± 0.08	7.15 ± 0.02
Hb (g/dL)	13.2 ± 0.1	15.3 ± 0.6^∗^	14.1 ± 0.5^♦^	12.9 ± 0.6	13.5 ± 1.0	12.2 ± 1.0

Blood gas analyses from arterial blood after 405 min of mechanical ventilation with FiO_2_ = 0.3. Data are shown as mean ± SD. Data from mice that died during the experiment were excluded. ^#^*p* < 0.001, sham-treated groups compared to TNF- and zVAD/TNF-treated groups; ^∗^*p* < 0.01 compared to WT sham and *p* < 0.05 compared to ASM^−/−^+TNF; ^♦^*p* < 0.01 compared to ASM^−/−^+zVAD/TNF.

## Data Availability

The data used to support the findings of this study are included within the article.

## Supplementary Materials

Supplementary Materials Supplemental Figure 1: a graphic depiction of the experimental design. Supplemental Figure 2: the acid sphingomyelinase activity in the lung, assessed by a radioactive ASM activity assay. Supplemental Figure 3: a cleaved caspase-3 immunostaining in the lungs.
