# Supplementary material for: Reevaluation of Lung Injury in TNF-Induced Shock: The Role of the Acid Sphingomyelinase
Source: Mediators Inflamm. 2020 May 1;2020:3650508. doi: 10.1155/2020/3650508 (PMC7211256; doi:10.1155/2020/3650508)
Supplement: Supplementary Materials — Supplemental Figure 1: a graphic depiction of the experimental design. Supplemental Figure 2: the acid sphingomyelinase activity in the lung, assessed by a radioactive ASM activity assay. Supplemental Figure 3: a cleaved caspase-3 immunostaining in the lungs. [file 3650508.f1.docx]

***Supplement***

***Fig. 1 Experimental design***


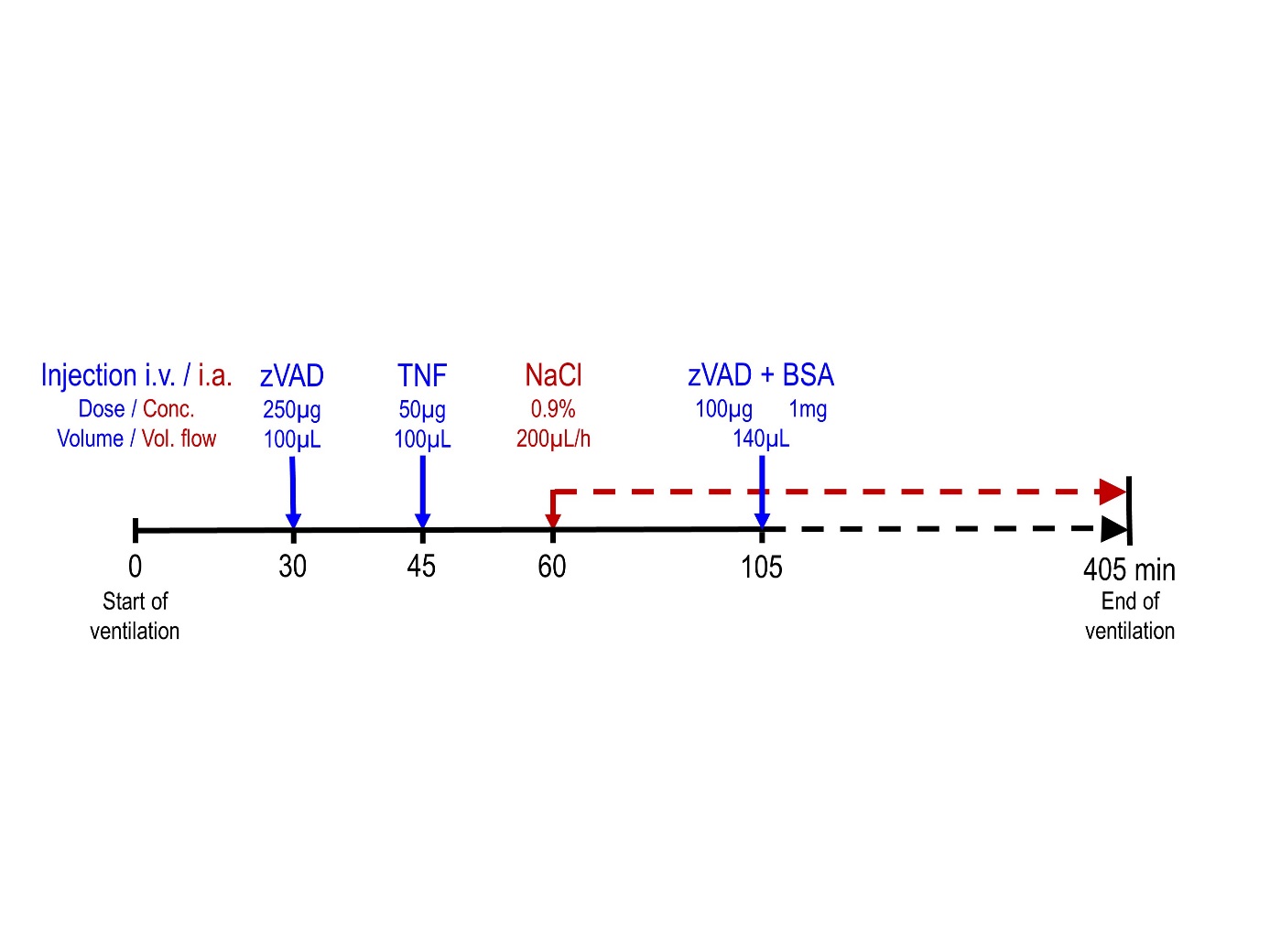


Mice were anaesthetized and connected to the FlexiVent ventilator. To ensure stable physiological base line values, 50 µg of TNF (in 100 µL) were injected intravenously after 45 min of ventilation. Mice were then ventilated for further 6 h. Half of the TNF-treated mice received 250µg (in 100 µL) of the caspase inhibitor zVAD 15 min before and 100µg zVAD (in 140 µL) together with 1mg bovine serum albumin (BSA) 60 min after TNF treatment. BSA was administered for assessment of microvascular permeability. All drugs were injected into the tail veins. Experiments were terminated after 405 minutes.

***Fig. 2*** ***Acid sphingomyelinase activity***


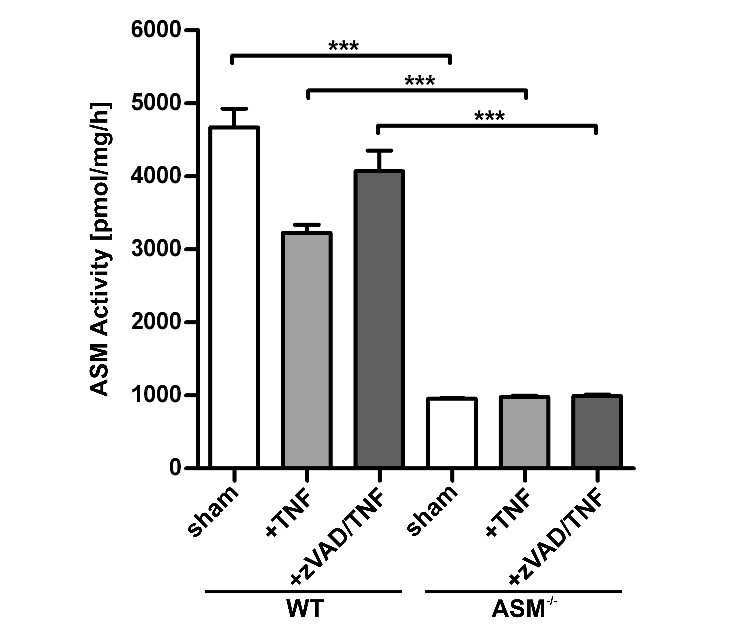


Lung tissue was subjected to a radioactive ASM activity assay. ASM activity was determined by using ^14^C-labelled sphingomyelin. For all samples, 10 μg protein were incubated with 40 μL substrate (73 nmol ^14^C-labelled sphingomyelin + 400 nmol sphingomyelin) at 37°C for 2 h. Lipids were separated by chloroform/methanol extraction, 4 mL scintillation liquid was added and radioactivity was counted in a β-counter. Activity values below 1000 pmol/mg/h are neglectable. All WT groups showed significant ASM activity in the lungs. Neither the treatment with TNF nor with zVAD/TNF had an influence on ASM activity in WT mice. Data are shown as mean + SEM. with WT sham n=5, WT+TNF n=5, WT+zVAD/TNF n=4, ASM^-/-^sham n=5, ASM^-/-^+TNF n=5 and ASM^-/-^+zVAD/TNF n=5. *** p < 0.001.

***Fig. 3*** ***Cleaved caspase-3 in the lung***


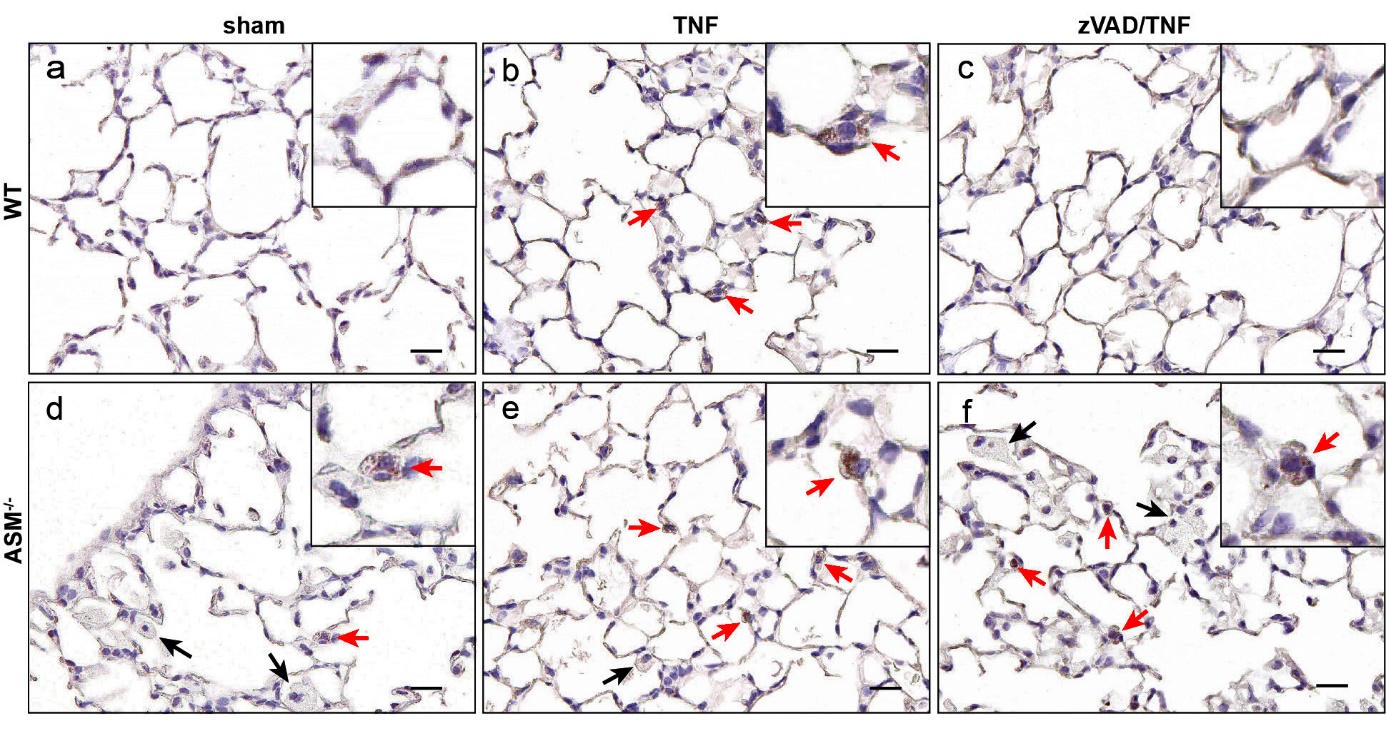


Paraffin embedded lungs were cut in sections of 3µm. The immunostaining was performed with an anti-cleaved-caspase-3 antibody (#9661, Cell Signaling Technology) and 3,3'-Diaminobenzidine, by following the manufacturers protocol. Sections were counterstained with hematoxylin. (a-c) Representative HE-stained sections from WT mice and (d-f) ASM^-/-^ mice, treated as indicated above the image. Black arrows indicate foam cells, red arrows indicate cleaved caspase-3 positive cells. Enlarged sections are shown in the upper right corners. Scale bars 20 µm, magnification 400x.
